# 3-Methyl-2-(4-methyl­phen­oxy)benzoic acid

**DOI:** 10.1107/S2414314623006004

**Published:** 2023-07-14

**Authors:** Kangkang Lu, Sihui Long

**Affiliations:** a School of Chemical Engineering and Pharmacy, Wuhan Institute of Technology, Wuhan, Hubei 430205, People’s Republic of China; University of Aberdeen, United Kingdom

**Keywords:** crystal structure, carb­oxy­lic acid inversion dimer

## Abstract

In the title compound, C_15_H_14_O_3_, the dihedral angle between the aromatic rings is 86.7 (9)°. In the crystal, carb­oxy­lic acid inversion dimers linked by pairwise O—H⋯O hydrogen bonds are formed.

## Abstract 2

The title compound features carb­oxy­lic acid inversion dimers in its extended structure.

## Graphical abstract

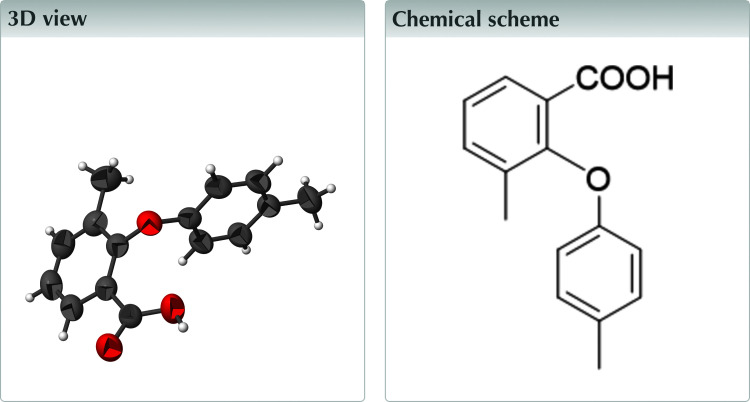

## Structure description

2-Phen­oxy­benzoic acids are isosteres of anthranilic acids that are potential anti-inflammatory drugs and conformationally flexible mol­ecules. Many anthranilic acids are polymorphic (Lopez-Mejias *et al.*, 2012 ▸; Sacchi, *et al.*, 2021 ▸). We wondered if 2-phen­oxy­benzoic acids would behave similarly to anthranilic acids in their polymorphism and as part of our work in this area, we now describe the synthesis and structure of the title compound, C_15_H_14_O_3_.

**Figure 1:**
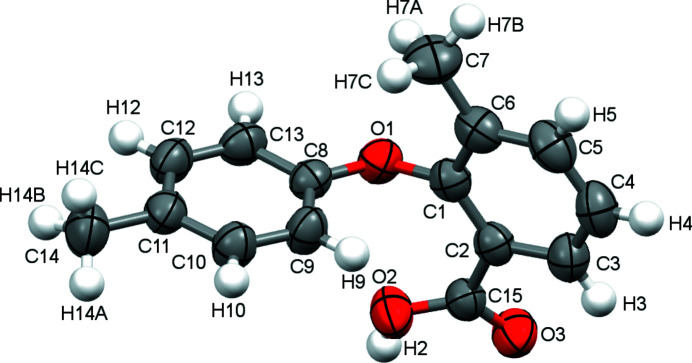
The mol­ecular structure of the title compound with displacement ellipsoids drawn at the 50% probability level.

**Figure 2:**
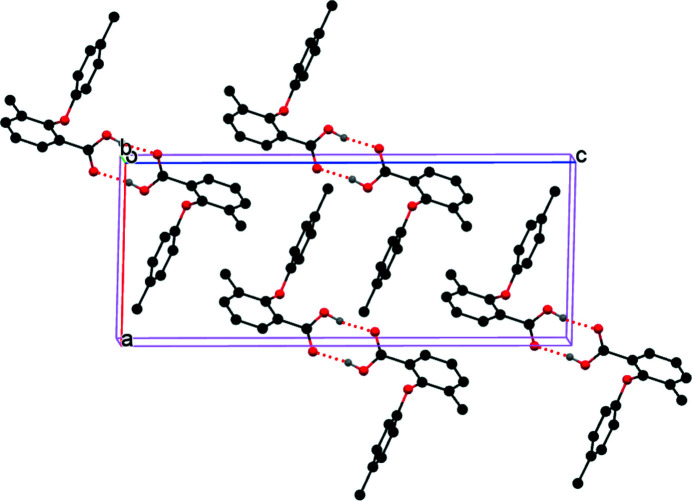
Packing of the mol­ecules in the title compound.

There is one mol­ecule in the asymmetric unit (Fig. 1 ▸). The mol­ecule has a nearly perpendicular conformation as evidenced by the dihedral angle between the benzoic acid ring and the phenol ring [86.7 (9)°]. In the crystal, the mol­ecules form carb­oxy­lic acid inversion dimers through pairwise O—H⋯O hydrogen bonds (Table 1 ▸, Fig. 2 ▸). Two weak C—H⋯π interactions are also observed.

## Synthesis and crystallization

**Figure 3:**
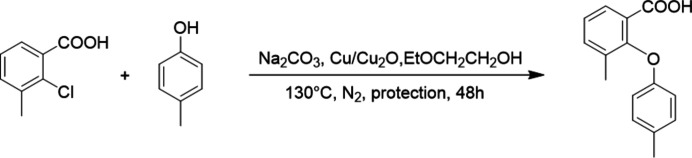
Reaction scheme.

**Figure 4:**
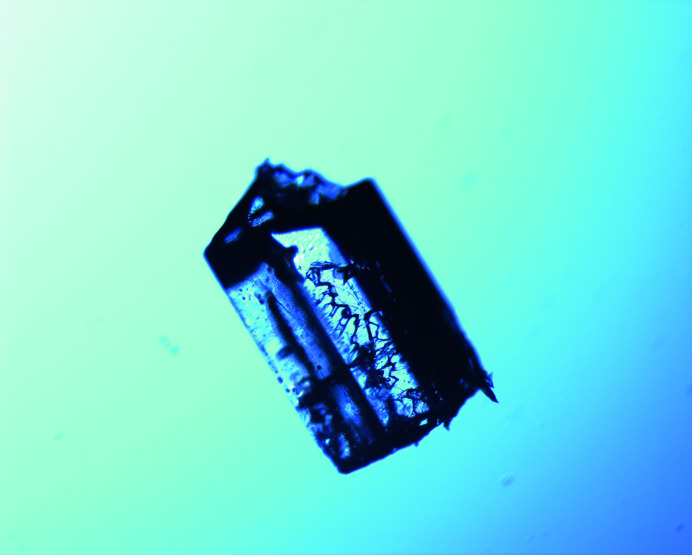
A representative crystal of the title compound.

The title compound was synthesized through an Ullmann reaction between 2-chloro-3-methyl-benzoic acid and 4-methyl­phenol (Fig. 3 ▸) in an effort to investigate the effect of substitution position and pattern on the solid-state behavior of 2-phen­oxy­benzoic acids. A pure sample was dissolved in ethyl acetate at 60°C. Then, the solution was cooled to room temperature and was allowed to evaporate slowly in a fume hood. Colorless block-shaped crystals (Fig. 4 ▸) were harvested after a week.

## Refinement

Crystal and refinement data are listed in Table 2 ▸.

## Supplementary Material

Crystal structure: contains datablock(s) I. DOI: 10.1107/S2414314623006004/hb4431sup1.cif

Structure factors: contains datablock(s) I. DOI: 10.1107/S2414314623006004/hb4431Isup2.hkl

Supporting information file. DOI: 10.1107/S2414314623006004/hb4431Isup3.cml

CCDC reference: 2280192

Additional supporting information:  crystallographic information; 3D view; checkCIF report

## Figures and Tables

**Table 1:** Hydrogen-bond geometry (Å, °)

*D*—H⋯*A*	*D*—H	H⋯*A*	*D*⋯*A*	*D*—H⋯*A*
O2—H2⋯O3^i^	0.86 (2)	1.80 (2)	2.6497 (14)	169 (3)
C4—H4⋯*Cg*1^ii^	0.93	2.92	3.6557 (18)	137
C5—H5⋯*Cg*2^iii^	0.93	2.97	3.8372 (15)	156

Symmetry codes: (i) 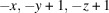 ; (ii) 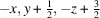 ; (iii) 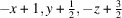 .

**Table 2:** Experimental details

Crystal data	
Chemical formula	C_15_H_14_O_3_
*M*_r_	242.26
Crystal system, space group	Monoclinic, *P*2_1_/*c*
Temperature (K)	293
*a*, *b*, *c* (Å)	8.95081 (17), 6.54110 (15), 21.9729 (4)
β (°)	91.2391 (18)
*V* (Å^3^)	1286.17 (5)
*Z*	4
Radiation type	Cu *K*α
μ (mm^−1^)	0.71
Crystal size (mm)	0.11 × 0.09 × 0.07

Data collection	
Diffractometer	Rigaku Oxford Diffraction, Synergy Custom system, HyPix
Absorption correction	Multi-scan (*CrysAlis PRO*; Rigaku OD, 2021 ▸)
*T*_min_, *T*_max_	0.856, 1.000
No. of measured, independent and observed [*I* > 2σ(*I*)] reflections	7830, 2527, 2197
*R*_int_	0.023
(sin θ/λ)_max_ (Å^−1^)	0.633

Refinement	
*R*[*F* ^2^ > 2σ(*F* ^2^)], *wR*(*F* ^2^), *S*	0.042, 0.124, 1.08
No. of reflections	2527
No. of parameters	169
No. of restraints	1
H-atom treatment	H atoms treated by a mixture of independent and constrained refinement
Δρ_max_, Δρ_min_ (e Å^−3^)	0.17, −0.17

Computer programs: *CrysAlis PRO* (Rigaku OD, 2021 ▸), *SHELXT* (Sheldrick, 2015*a* ▸), *SHELXL2018/3* (Sheldrick, 2015*b* ▸) and *OLEX2* (Dolomanov *et al.*, 2009 ▸).

## full crystallographic data

### Crystal data

**Table:**

C_15_H_14_O_3_	*F*(000) = 512
*M**_r_* = 242.26	*D*_x_ = 1.251 Mg m^−^^3^
Monoclinic, *P*2_1_/*c*	Cu *K*α radiation, λ = 1.54184 Å
*a* = 8.95081 (17) Å	Cell parameters from 5741 reflections
*b* = 6.54110 (15) Å	θ = 4.0–76.9°
*c* = 21.9729 (4) Å	µ = 0.71 mm^−^^1^
β = 91.2391 (18)°	*T* = 293 K
*V* = 1286.17 (5) Å^3^	Block, colourless
*Z* = 4	0.11 × 0.09 × 0.07 mm

### Data collection

**Table:**

Rigaku Oxford Diffraction, Synergy Custom system, HyPix diffractometer	2527 independent reflections
Radiation source: Rotating-anode X-ray tube, Rigaku (Cu) X-ray Source	2197 reflections with *I* > 2σ(*I*)
Mirror monochromator	*R*_int_ = 0.023
Detector resolution: 10.0000 pixels mm^-1^	θ_max_ = 77.6°, θ_min_ = 4.0°
ω scans	*h* = −11→11
Absorption correction: multi-scan (CrysalisPro; Rigaku OD, 2021)	*k* = −8→8
*T*_min_ = 0.856, *T*_max_ = 1.000	*l* = −27→20
7830 measured reflections	

### Refinement

**Table:**

Refinement on *F*^2^	Primary atom site location: dual
Least-squares matrix: full	Hydrogen site location: mixed
*R*[*F*^2^ > 2σ(*F*^2^)] = 0.042	H atoms treated by a mixture of independent and constrained refinement
*wR*(*F*^2^) = 0.124	*w* = 1/[σ^2^(*F*_o_^2^) + (0.0665*P*)^2^ + 0.1182*P*] where *P* = (*F*_o_^2^ + 2*F*_c_^2^)/3
*S* = 1.08	(Δ/σ)_max_ = 0.001
2527 reflections	Δρ_max_ = 0.17 e Å^−^^3^
169 parameters	Δρ_min_ = −0.17 e Å^−^^3^
1 restraint	

### Special details

**Table:**

Geometry. All esds (except the esd in the dihedral angle between two l.s. planes) are estimated using the full covariance matrix. The cell esds are taken into account individually in the estimation of esds in distances, angles and torsion angles; correlations between esds in cell parameters are only used when they are defined by crystal symmetry. An approximate (isotropic) treatment of cell esds is used for estimating esds involving l.s. planes.
Refinement. The C-bound H atoms were placed geometrically (C—H = 0.93–0.96 Å) and refined as riding atoms.

### Fractional atomic coordinates and isotropic or equivalent isotropic displacement parameters (Å^2^)

**Table:**

	*x*	*y*	*z*	*U*_iso_*/*U*_eq_	
O1	0.24620 (10)	0.17742 (14)	0.64109 (4)	0.0593 (3)	
O2	0.15526 (12)	0.4152 (2)	0.54426 (5)	0.0769 (3)	
H2	0.110 (3)	0.422 (5)	0.5094 (8)	0.190 (13)*	
O3	−0.05029 (11)	0.58182 (18)	0.56942 (4)	0.0725 (3)	
C1	0.21476 (13)	0.3529 (2)	0.67392 (6)	0.0536 (3)	
C2	0.12444 (13)	0.5035 (2)	0.64797 (6)	0.0541 (3)	
C3	0.08109 (16)	0.6693 (2)	0.68345 (7)	0.0656 (4)	
H3	0.018988	0.769656	0.666817	0.079*	
C4	0.13026 (18)	0.6842 (3)	0.74310 (7)	0.0745 (4)	
H4	0.102602	0.795630	0.766625	0.089*	
C5	0.22069 (17)	0.5335 (3)	0.76790 (6)	0.0728 (4)	
H5	0.253775	0.546005	0.808144	0.087*	
C6	0.26370 (15)	0.3642 (2)	0.73468 (6)	0.0639 (4)	
C7	0.3572 (2)	0.1978 (3)	0.76307 (8)	0.0875 (5)	
H7A	0.324401	0.067562	0.747699	0.131*	
H7B	0.347063	0.200889	0.806463	0.131*	
H7C	0.460117	0.218413	0.753209	0.131*	
C8	0.38771 (14)	0.15863 (19)	0.61722 (6)	0.0531 (3)	
C9	0.48515 (15)	0.3192 (2)	0.61186 (7)	0.0643 (4)	
H9	0.458835	0.448913	0.625247	0.077*	
C10	0.62304 (16)	0.2860 (2)	0.58630 (7)	0.0672 (4)	
H10	0.688959	0.395109	0.582847	0.081*	
C11	0.66564 (15)	0.0951 (2)	0.56573 (6)	0.0624 (3)	
C12	0.56571 (17)	−0.0629 (2)	0.57189 (7)	0.0676 (4)	
H12	0.591697	−0.192455	0.558302	0.081*	
C13	0.42802 (17)	−0.0346 (2)	0.59764 (6)	0.0634 (4)	
H13	0.362890	−0.144278	0.601829	0.076*	
C14	0.81667 (19)	0.0625 (3)	0.53797 (9)	0.0852 (5)	
H14A	0.844404	0.182635	0.515791	0.128*	
H14B	0.811909	−0.052342	0.510806	0.128*	
H14C	0.889706	0.036619	0.569672	0.128*	
C15	0.07176 (14)	0.4993 (2)	0.58337 (6)	0.0554 (3)	

### Atomic displacement parameters (Å^2^)

**Table:**

	*U*^11^	*U*^22^	*U*^33^	*U*^12^	*U*^13^	*U*^23^
O1	0.0555 (5)	0.0529 (5)	0.0694 (6)	−0.0043 (4)	−0.0001 (4)	−0.0034 (4)
O2	0.0720 (6)	0.1022 (9)	0.0567 (6)	0.0235 (6)	0.0036 (5)	−0.0043 (5)
O3	0.0633 (6)	0.0874 (8)	0.0666 (6)	0.0174 (5)	−0.0013 (4)	0.0021 (5)
C1	0.0477 (6)	0.0558 (7)	0.0573 (7)	−0.0064 (5)	0.0040 (5)	−0.0015 (5)
C2	0.0488 (6)	0.0583 (7)	0.0556 (6)	−0.0038 (5)	0.0058 (5)	−0.0010 (5)
C3	0.0608 (8)	0.0676 (9)	0.0689 (8)	0.0038 (6)	0.0092 (6)	−0.0065 (7)
C4	0.0707 (9)	0.0826 (11)	0.0707 (9)	−0.0025 (7)	0.0111 (7)	−0.0222 (8)
C5	0.0674 (8)	0.0947 (12)	0.0562 (7)	−0.0093 (8)	0.0003 (6)	−0.0114 (7)
C6	0.0559 (7)	0.0769 (9)	0.0587 (7)	−0.0078 (6)	−0.0021 (6)	0.0025 (6)
C7	0.0902 (11)	0.0955 (13)	0.0758 (10)	0.0046 (9)	−0.0197 (9)	0.0106 (9)
C8	0.0541 (6)	0.0514 (7)	0.0536 (6)	0.0022 (5)	−0.0039 (5)	0.0015 (5)
C9	0.0614 (7)	0.0487 (7)	0.0832 (9)	0.0010 (6)	0.0074 (6)	−0.0071 (6)
C10	0.0609 (8)	0.0582 (8)	0.0826 (9)	−0.0010 (6)	0.0072 (7)	−0.0027 (7)
C11	0.0651 (8)	0.0622 (8)	0.0599 (7)	0.0098 (6)	0.0012 (6)	−0.0003 (6)
C12	0.0807 (9)	0.0523 (8)	0.0698 (8)	0.0117 (7)	0.0016 (7)	−0.0061 (6)
C13	0.0735 (8)	0.0481 (7)	0.0683 (8)	−0.0023 (6)	−0.0031 (6)	−0.0007 (6)
C14	0.0781 (10)	0.0813 (11)	0.0969 (12)	0.0168 (9)	0.0168 (9)	−0.0063 (9)
C15	0.0523 (6)	0.0560 (7)	0.0581 (7)	0.0014 (5)	0.0052 (5)	0.0013 (5)

### Geometric parameters (Å, º)

**Table:**

O1—C1	1.3875 (15)	C7—H7B	0.9600
O1—C8	1.3868 (15)	C7—H7C	0.9600
O2—H2	0.860 (10)	C8—C9	1.3719 (19)
O2—C15	1.2759 (16)	C8—C13	1.3857 (18)
O3—C15	1.2508 (16)	C9—H9	0.9300
C1—C2	1.3890 (19)	C9—C10	1.3841 (19)
C1—C6	1.3980 (18)	C10—H10	0.9300
C2—C3	1.3957 (19)	C10—C11	1.385 (2)
C2—C15	1.4862 (18)	C11—C12	1.375 (2)
C3—H3	0.9300	C11—C14	1.510 (2)
C3—C4	1.377 (2)	C12—H12	0.9300
C4—H4	0.9300	C12—C13	1.380 (2)
C4—C5	1.380 (2)	C13—H13	0.9300
C5—H5	0.9300	C14—H14A	0.9600
C5—C6	1.386 (2)	C14—H14B	0.9600
C6—C7	1.501 (2)	C14—H14C	0.9600
C7—H7A	0.9600		
			
C8—O1—C1	117.85 (9)	C9—C8—C13	120.10 (12)
C15—O2—H2	108 (2)	C13—C8—O1	116.38 (11)
O1—C1—C2	119.74 (11)	C8—C9—H9	120.4
O1—C1—C6	118.51 (12)	C8—C9—C10	119.27 (13)
C2—C1—C6	121.47 (12)	C10—C9—H9	120.4
C1—C2—C3	119.22 (12)	C9—C10—H10	119.1
C1—C2—C15	123.29 (12)	C9—C10—C11	121.88 (14)
C3—C2—C15	117.49 (12)	C11—C10—H10	119.1
C2—C3—H3	120.0	C10—C11—C14	120.94 (15)
C4—C3—C2	120.00 (14)	C12—C11—C10	117.47 (13)
C4—C3—H3	120.0	C12—C11—C14	121.59 (14)
C3—C4—H4	120.1	C11—C12—H12	119.0
C3—C4—C5	119.85 (14)	C11—C12—C13	121.92 (13)
C5—C4—H4	120.1	C13—C12—H12	119.0
C4—C5—H5	119.0	C8—C13—H13	120.3
C4—C5—C6	122.02 (13)	C12—C13—C8	119.36 (13)
C6—C5—H5	119.0	C12—C13—H13	120.3
C1—C6—C7	121.27 (14)	C11—C14—H14A	109.5
C5—C6—C1	117.42 (14)	C11—C14—H14B	109.5
C5—C6—C7	121.30 (14)	C11—C14—H14C	109.5
C6—C7—H7A	109.5	H14A—C14—H14B	109.5
C6—C7—H7B	109.5	H14A—C14—H14C	109.5
C6—C7—H7C	109.5	H14B—C14—H14C	109.5
H7A—C7—H7B	109.5	O2—C15—C2	118.22 (11)
H7A—C7—H7C	109.5	O3—C15—O2	122.84 (12)
H7B—C7—H7C	109.5	O3—C15—C2	118.92 (11)
C9—C8—O1	123.51 (11)		
			
O1—C1—C2—C3	173.51 (11)	C3—C4—C5—C6	−0.5 (2)
O1—C1—C2—C15	−7.17 (18)	C4—C5—C6—C1	1.5 (2)
O1—C1—C6—C5	−174.93 (12)	C4—C5—C6—C7	−177.62 (15)
O1—C1—C6—C7	4.16 (19)	C6—C1—C2—C3	−0.29 (19)
O1—C8—C9—C10	−178.73 (12)	C6—C1—C2—C15	179.03 (12)
O1—C8—C13—C12	178.25 (12)	C8—O1—C1—C2	105.42 (13)
C1—O1—C8—C9	−15.62 (18)	C8—O1—C1—C6	−80.60 (14)
C1—O1—C8—C13	165.09 (11)	C8—C9—C10—C11	0.2 (2)
C1—C2—C3—C4	1.3 (2)	C9—C8—C13—C12	−1.1 (2)
C1—C2—C15—O2	−30.72 (19)	C9—C10—C11—C12	−0.4 (2)
C1—C2—C15—O3	150.82 (13)	C9—C10—C11—C14	179.97 (14)
C2—C1—C6—C5	−1.06 (19)	C10—C11—C12—C13	−0.2 (2)
C2—C1—C6—C7	178.03 (13)	C11—C12—C13—C8	0.9 (2)
C2—C3—C4—C5	−0.9 (2)	C13—C8—C9—C10	0.5 (2)
C3—C2—C15—O2	148.61 (14)	C14—C11—C12—C13	179.47 (14)
C3—C2—C15—O3	−29.85 (18)	C15—C2—C3—C4	−178.07 (13)

### Hydrogen-bond geometry (Å, º)

**Table:**

*D*—H···*A*	*D*—H	H···*A*	*D*···*A*	*D*—H···*A*
O2—H2···O3^i^	0.86 (2)	1.80 (2)	2.6497 (14)	169 (3)
C4—H4···*Cg*1^ii^	0.93	2.92	3.6557 (18)	137
C5—H5···*Cg*2^iii^	0.93	2.97	3.8372 (15)	156

Symmetry codes: (i) −*x*, −*y*+1, −*z*+1; (ii) −*x*, *y*+1/2, −*z*+3/2; (iii) −*x*+1, *y*+1/2, −*z*+3/2.
